# The Association between Self-Care Activities and Depression in Adult Patients with Type 2 Diabetes in Saudi Arabia: A Cross-Sectional Web-Based Survey Study

**DOI:** 10.3390/jcm13020419

**Published:** 2024-01-12

**Authors:** Sawsan M. Kurdi, Ahmad Alamer, Aya Albaggal, Marwa Alsuwaiket, Fawaz M. Alotaibi, Ibrahim M. Asiri, Dhfer M. Alshayban, Mohammed M. Alsultan, Bashayer Alshehail, Bassem A. Almalki, Dania Hussein, Mansour M. Alotaibi, Osamah M. Alfayez

**Affiliations:** 1 Department of Pharmacy Practice, College of Clinical Pharmacy, Imam Abdulrahman bin Faisal University, Dammam 34221, Saudi Arabia; 2190004868@iau.edu.sa (A.A.); 2190001834@iau.edu.sa (M.A.); fmalotaibi@iau.edu.sa (F.M.A.); imasiri@iau.edu.sa (I.M.A.); dmalshayban@iau.edu.sa (D.M.A.); mmaalsultan@iau.edu.sa (M.M.A.); bmalshehail@iau.edu.sa (B.A.); baalmalki@iau.edu.sa (B.A.A.); 2 Department of Clinical Pharmacy, Prince Sattam bin Abdulaziz University, Alkharj 16273, Saudi Arabia; aa.alamer@psau.edu.sa; 3 Department of Pharmacology, College of Clinical Pharmacy, Imam Abdulrahman bin Faisal University, Dammam 34221, Saudi Arabia; dahussein@iau.edu.sa; 4 Pharmacy Practice Department, College of Clinical Pharmacy, King Faisal University, Al-Ahsa 31982, Saudi Arabia; mmqalotaibi@kfu.edu.sa; 5 Department of Pharmacy Practice, College of Pharmacy, Qassim University, Qassim 51911, Saudi Arabia; oalfayez@qu.edu.sa

**Keywords:** depression, diabetes, type 2 diabetes, self-care, cross-sectional study, survey

## Abstract

This study examined the level of adherence to self-care behaviors among individuals with type 2 diabetes in Saudi Arabia and its connection with depression and demographic factors. A cross-sectional survey was conducted among diabetes patients using the Patient Health Questionnaire (PHQ-9) to measure depression and the Summary of Diabetes Self-Care Activities (SDSCA) to evaluate diabetes self-care activities. Among the 252 participants who completed the survey, 43.2% were older than 55 and 59% were men. The ordinal regression model showed an association between the PHQ-9 and SDSCA scores with an OR of 0.83 (95% CI: 0.71 to 0.96, *p* = 0.013). The PHQ-9 score was significantly associated with blood sugar monitoring (OR: 0.90 [95% CI: 0.82 to 0.99, *p* = 0.003]), exercise (OR: 0.88 [95% CI: 0.79 to 0.98, *p* = 0.002]), and diet (OR: 0.94 [95% CI: 0.85 to 1.03, *p* = 0.045]). Of all the diabetes-related factors, only a history of hospitalization and receiving diabetes education were found to be associated with improved self-care behaviors. In conclusion, a negative association was found between PHQ-9 scores and the SDSCA mean score and most daily diabetic self-care behavior components.

## 1. Introduction

Diabetes is a chronic disease that is characterized by elevated blood glucose levels. It is a global health concern that has witnessed a significant rise in prevalence over the past few decades. The International Diabetes Federation’s (IDF) *Diabetes Atlas*, 10th edition, documents an ongoing rise in diabetes prevalence worldwide, posing an ever-growing burden on global health and well-being. The global prevalence of diabetes in people between the ages of 20 and 79 years is estimated to increase to 12.2% by 2045 compared to 10.5% in 2021. Diabetes appears to be most prevalent in those between the ages of 75 and 79 years, with comparable patterns of incidence between both genders. According to a recent IDF report, the prevalence of diabetes in adults in Saudi Arabia is approximately 17.7%, which translates to approximately 4.2 million cases of diabetes in adults in Saudi Arabia alone [1].

Those with uncontrolled diabetes are prone to the development of complications that significantly deteriorate the quality of life of affected individuals. Such complications often appear at neurological, macrovascular, and microvascular levels, culminating in cardiovascular disease, renal disease, neurological damage, and retinopathies. Self-care activities are an effective preventive measure for the management of diabetes and the prevention of disease-related complications. Diabetes-related self-care activities (DRSCAs) are embraced by many who view them as the cornerstone of effective diabetes management. Physical activity, healthy eating, blood glucose monitoring, good problem-solving skills, medication adherence, healthy coping and stress management skills, and risk-reduction behaviors are seven essential self-care behaviors that can help predict good outcomes in patients with diabetes [2]. The importance of diabetes self-management in patients with type 2 diabetes cannot be underestimated. It has been found that self-care practices have a positive impact on long-term disease management, including glycemic control, reduced complications, and improvement in the quality of life. Consequently, quality of life correlates with self-care practices and the capacity for self-care compromise [3]. On the other hand, while there may be many patients with diabetes and well-managed disease status who do not adhere to self-care activities, their mortality and quality of life may ultimately be negatively impacted by their lack of self-care behaviors [4]. Furthermore, self-management empowers individuals with diabetes to develop skills and knowledge that enable them to feel in control of their condition. Hence, diabetic self-care methods are globally acknowledged as the backbone of effective diabetes control [5].

Some studies have found that people with diabetes may experience difficulty in applying multiple self-care behaviors concurrently in a consistent manner. Mental illness—notably depression—is one of the factors that appears to have a significant impact on diabetes self-care practice in patients with type 2 diabetes (T2D) [6]. It has been estimated that depression affects one in four patients with diabetes [7]. Studies from Saudi Arabia found that depression ranged from 29.4% to 49.6% in those with T2D [8,9,10]. Studies from neighboring countries have found an even higher prevalence of depression. For instance, a Jordanian study found depression symptoms to be present in 76% of patients [11].

Several studies have explored the relationship between depression and self-care behaviors in individuals with diabetes. Patients with diabetes and depression are less likely to adhere to self-care, tending to miss their doses more frequently and subsequently experiencing a higher incidence of complications, poor outcomes, and poor quality of life. A study by Gonzalez et al. found that depression is a categorical risk factor for nonadherence and suggested that interventions aimed at alleviating depressive symptoms could lead to significant improvements in diabetes self-care [12]. Another study conducted in Taiwan similarly found that depression was negatively associated with self-care behavior [13]. It is important to routinely assess patients for diabetes distress and psychosocial issues impacting their diabetes self-management. The clinical guidelines encourage patients with diabetes to be screened for depression annually, at diagnoses of complications, or when there is a significant change in their medical status [14].

While many studies have investigated the relationship between depression and diabetes self-care globally, research focused on the Saudi Arabian context is limited. Given Saudi Arabia’s high prevalence of diabetes, there is a pressing need for localized research. This study aimed to investigate the level of adherence to self-care behaviors among people with type 2 diabetes in Saudi Arabia and its association with depression, self-care behavior, and demographic factors.

## 2. Methods

### 2.1. Study Design, Setting, and Sample Selection

This was a cross-sectional study of diabetes patients surveyed between January and April 2023. The sample population for the survey was propagated through social media via snowball methodology. Our inclusion criteria were patients with a type 2 diabetes diagnosis, aged 18 years old or over, residing in Saudi Arabia, and consenting to participate in the survey. The study was approved by Imam Abdulrahman University’s (IAU) ethics committee (IRB no. 2-22-05-504).

### 2.2. Survey Instruments

The Patient Health Questionnaire (PHQ-9), a well-known validated tool, was used to measure depression severity [15]. The PHQ-9 score consists of nine items that assess patients over a 14-day period. To assess each item, patients answer on a scale ranging from 0 to 3, where 0 = ‘not at all’, 1 = ‘several days’, 2 = ‘more than half the days’, and 3 = ‘nearly every day’. The total score (a continuous variable) ranges from 0 to 27. The Arabic-validated PHQ-9 instrument was used in this study [16].

Another tool used in the survey to measure diabetes self-care activities was the Summary of Diabetes Self-Care Activities (SDSCA) [17]. The summary score asks patients to answer how many days in the past 7 days they engaged in performing self-care activities with the following components: diet, medication, exercise, blood sugar checks, foot care, and smoking. A higher score indicates greater engagement with diabetes self-care activities. The SDSCA has been validated and translated into many languages, including Arabic [18].

### 2.3. Outcomes and Sample Size

The primary point of investigation was to measure the association between the PHQ-9 score and the SDSCA score. Secondarily, we investigated the association between the PHQ-9 score and individual components of the SDSCA. Additionally, we explored factors affecting the SDSCA scores. To detect a simple correlation of r = 0.2 using a two-sided test with a 5% significance level (α = 0.05) and 80% power (β = 0.2), the required sample size was approximately 194 subjects.

### 2.4. Statistical Analysis

Descriptive statistics were used to summarize the data, which included means with standard deviations or medians with their 25th and 75th percentiles for continuous variables. We presented the data visually as box plots. For the categorical variables, we used frequencies and percentages. To analyze our primary outcome, we utilized ordinal regression models. The ordinal regression model is an extension of the Wilcoxon–Mann–Whitney–Kruskal–Wallis–Spearman model [19]. The semi-parametric model assumes nothing about the shape of the Y variable (outcome). It can work with discrete, continuous, and discontinuous variables, and it is robust against outliers, which makes it suitable for our analysis [20]. We assumed the PHQ-9 score as the dependent variable and the SDSCA score as the independent variable. The proportionality assumption, if not met, is not fatal to the analysis. We used such a model because it allows for analyzing ordered categorical dependent variables—in our case, the PHQ-9 score (higher values indicate worse cases) while considering multiple independent variables. To identify potential confounders, we utilized directed acyclic diagrams (DAGs). In this method, researchers draw their hypotheses and assumptions about the data-generating process in which variables influence each other, creating causal pathways. This transparent approach allows us to isolate a sufficient adjustment set to obtain unbiased estimates of the true total causal effect of the variable of interest on the outcome. In addition, this method identifies collider variables, a non-causal pathway in which controlling for such variables will create a biased association. A mediator variable, a variable that mediates the effect between two variables of interest, should be avoided to condition on when the total causal effect is of interest [21]. Head arrows are drawn between variables of interest to indicate potential causal relationships. Confounder variables are drawn in red circles as they influence both the independent variable of interest and the outcome variable. Conditioning on these confounders can isolate the total causal effect. The strength of the relationship between PHQ-9 and SDSCA was tested using ordinal regression and we presented the coefficients as odds ratios. Odds ratios of <1 indicate an association of a less severe depression score with an increasing SDSCA and vice versa. The degree of certainty of these estimates was bound by 95% confidence intervals. Results were considered statistically significant at a *p*-value < 0.05. As for the exploratory analysis, we utilized univariable and multivariable ordinal regression models. All analyses were conducted using the Jamovi open-source statistical software (Version 2.4.1, The Jamovi Project, 2023).

## 3. Results

A total of 252 participants completed the survey, of whom 59% were male and 43.2% were aged over 55 years old. Most participants were married (81%) and had a high school educational level and above (61%). Additionally, 36.3% were employed and 39.8% were retired. Other demographic characteristics are described in Table 1. Around 40% of the patients had had diabetes for more than ten years, 25% for five to ten years, and 35% for less than five years. Regarding diabetes treatment, more than a third of the patients were on oral hypoglycemic medications (42%), and approximately 20% were on combinations of oral hypoglycemic medications and insulin. Most patients had comorbidities (95%), including dyslipidemia and hypertension (45% each), other cardiovascular diseases (8%), asthma or other respiratory diseases (8%), and chronic renal disease (1%). Furthermore, most patients had diabetes-related complications (78%) and 80% had a history of hospitalization. Only 29% of the patients had received diabetes education.

### 3.1. Depression

**Figure 1:**
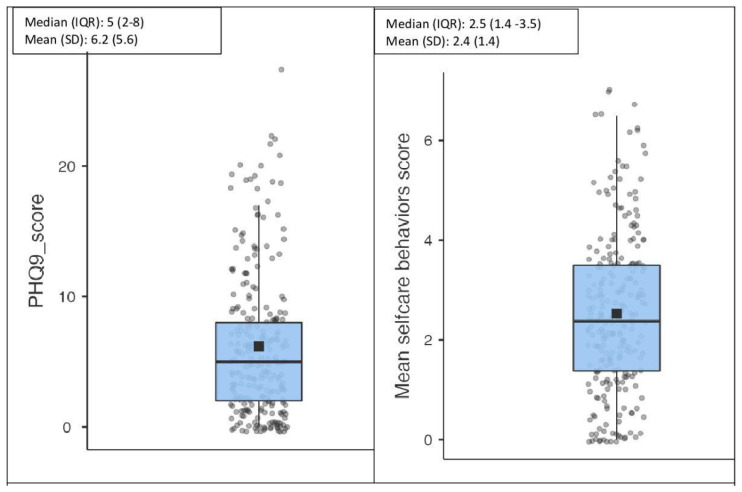
Boxplot description of the dispersion of PHQ-9 and self-care activity scores in the sample.

Depressive symptoms were prevalent in the study sample: 3% had severe depressive symptoms, 7% had moderate to severe symptoms, 11% had moderate symptoms, and 33% had mild depressive symptoms. The median (interquartile range, IQR) PHQ-9 score was five (IQR: 2–8). This is illustrated in a box plot in Figure 1.

### 3.2. Self-Care Activities

The total median (IQR) number of days used for self-care activities was 2.5 (IQR: 1.4–3.5 days). The dispersion of the score is illustrated in a box plot in Figure 1. Diet activities were performed on 3.0 median days (IQR: 0.5–5 days), and blood glucose monitoring was performed on 2.5 median days (IQR: 0–5.5 days). Exercise and foot examinations were performed on 0.5 median days (IQR: 0–3 days).

### 3.3. The Association between Depression and Self-Care Activities

**Figure 2:**
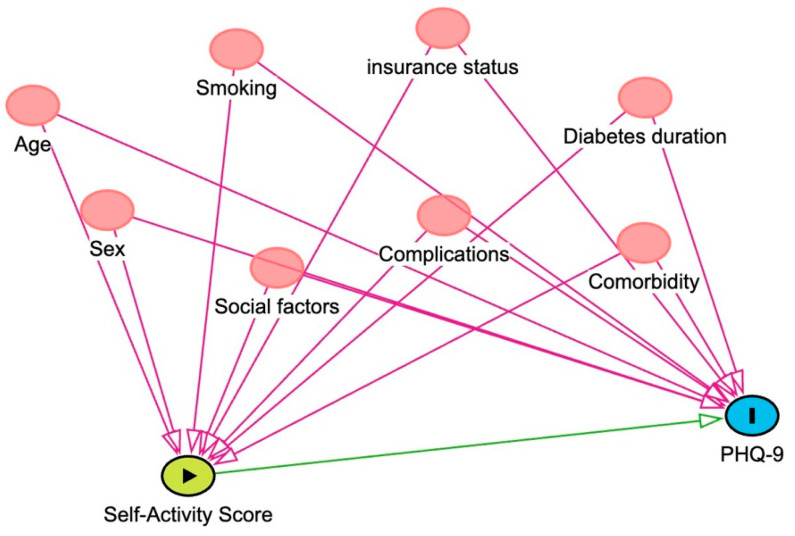
Directed acyclic diagrams (DAGs) illustrating the relationship between the variables to estimate the total causal effect. Spots in red represent potential confounders that have a relationship with both the outcome and our independent variable of interest.

**Figure 3:**
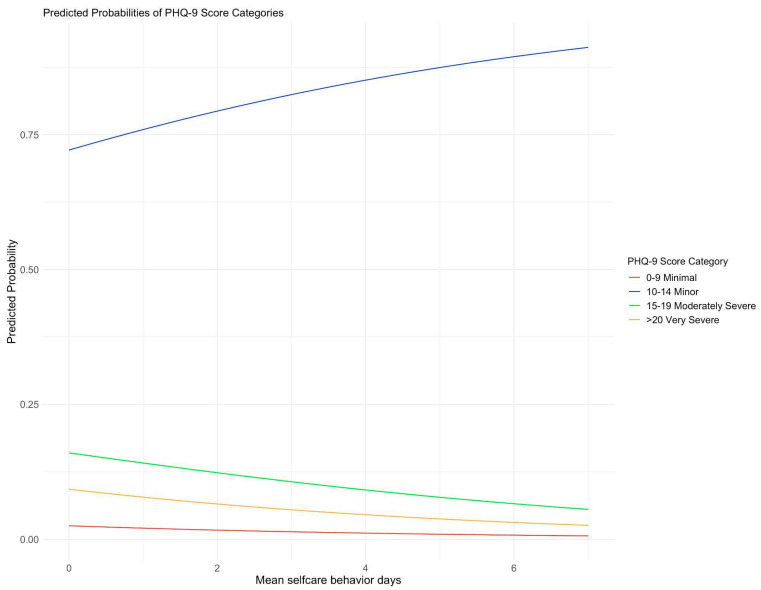
Partial effect plot that illustrates how the probabilities of falling into each category of PHQ-9 score change as the variable of mean self-care behavior days changes. The effect was adjusted for age, sex, social factors, insurance, diabetes duration, complications, smoking status, and comorbid conditions. The colors of the lines are used to distinguish between the different categories of PHQ-9 scores. If a line corresponding to a category is moving upward, it indicates that as the mean days spent on self-care behaviors increase, the predicted probability of falling into that category also increases.

To illustrate the potential confounders, DAGs are drawn in Figure 2. The association between self-care activities and depression is presented in Table 2. When adjusted for potential confounders, the ordinal regression model shows an association between the two variables with an odds ratio (OR) of 0.83 (95% CI: 0.71 to 0.96, *p* = 0.013). To illustrate this relationship, the predicted probabilities of PHQ-9 for each category were drawn as the mean self-care activity changes (Figure 3).

When assessed separately, diet (OR: 0.94 [95% CI: 0.85 to 1.03, *p* = 0.045]), blood sugar monitoring (OR: 0.90 [95% CI: 0.82 to 0.99, *p* = 0.003]), and exercise (OR: 0.88 [95% CI: 0.79 to 0.98, *p* = 0.002]) were significantly associated with the PHQ-9 score.

### 3.4. Factors Associated with Diabetes Self-Care Activities

In the multivariable analysis, of all the diabetes-related factors, only a history of hospitalization (OR: 1.81 [95% CI: 1.03 to 3.19, *p* = 0.039]), receiving diabetes education (OR: 1.89 [95% CI: 1.09 to 3.29, *p* = 0.024]), and having asthma or other respiratory diseases (OR: 0.40 [95% CI: 0.17 to 0.94, *p* = 0.035]) were found to be associated with improvements in self-care behaviors.

## 4. Discussion

The key finding in the current study is a statistically significant negative association between PHQ-9 scores and the mean score of the SDSCA. With each average day increase in the SDSCA score, there was a 17% reduction in the odds of higher PHQ-9 scores. These findings are visually represented in Figure 3, where individuals with an average of 6 days of SDCA had a higher probability of falling within the 10–14 minor PHQ-9 score category. Furthermore, our analysis revealed that individual components of self-care activities, such as diet, blood sugar monitoring, and exercise, were significantly associated with PHQ-9 scores. The exception was foot care, which did not show a significant association after adjusting for confounders. Self-care is a vital component of managing type 2 diabetes, and understanding the factors that can correlate negatively or positively with diabetes self-care activities is essential [3]. Patients with chronic diseases like type 2 diabetes are at an increased risk of experiencing depression, which can adversely affect their ability to manage their condition [7]. Our study reaffirms the association between depression and diabetes self-care activities, including the individual components of these activities.

In terms of self-care activities, our data revealed that the median (IQR) days spent on self-care activities was 2.5 days (IQR: 1.4–3.5 days), which is lower than reported in other studies [8,11,22,23,24,25,26]. This indicates very poor self-care activities among type 2 diabetes patients in Saudi Arabia. All individual components of self-care activities addressed in this study were poorly performed, with exercise and foot exams being the least performed. Physical activity is usually a challenge for patients with type 2 diabetes. Many factors can play a role in lower adherence to exercise. Specifically, Saudi Arabia and some neighboring countries face challenges to outdoor physical activity due to the hot weather most of the year. Although exercise has been proven to be beneficial in helping to delay and manage type 2 diabetes [27,28], patients’ involvement remains low [11,22,23,24,25,26]. The people of Saudi Arabia are no exception to this situation, even though there have been some reports indicating that recently more people in Saudi Arabia have begun engaging in physical activities, as illustrated by an analysis of national survey data [22].

Surprisingly, the daily foot exam was among the least performed activities (median of 0.5 days). In Saudi culture, most patients perform daily prayers which begin with ablutions (*Wudu*), which involve washing their feet. This result does not coincide with other studies conducted in Saudi Arabia and is challenging to elucidate in light of the nation’s religious and cultural practices. Alhiti et al. reported that the mean adherence score for foot care was 3.28 days [24]. Another study found a mean of 2.6 days [22], while Aljohani et al. reported a mean of 2.72 days [23]. Similarly, studies in neighboring countries with predominantly Muslim populations (Jordan and Kuwait) found higher averages of 2.4 days and 4.3 days, respectively [11,25]. The exact reason why we have observed such low adherence is not entirely clear. This might be partly due to the number of items in the survey as some studies included two items pertinent to foot care while others included five items. Moreover, the way the survey was conducted (face-to-face vs. online) might have played a role in the variations in the results reported. All the previously mentioned studies, including ours, have utilized the same validated tool (the SDSCA). It is important to acknowledge that the questionnaire utilized in our research comprises two items that pertain to foot care. The first item asks how many times the patient checks their feet, while the second item asks how often the patient checks the interior of their shoes. While patients are expected to inspect their feet daily as previous literature reported, they may not check the interior of their shoes, which could affect total foot care adherence, leading to the low average of days invested in foot care observed. Our study highlights the importance of patient education regarding foot care, which should be carried out more often by healthcare providers and diabetes educators in diabetes clinics.

It is important to note that the Arabic version of the self-care activity questionnaire distributed to patients did not ask patients about medication use. Usually, patient adherence to taking medications, especially oral medications, ranks among the top-performed daily activities by patients. For instance, studies conducted in Saudi Arabia found that patients take their medication properly most of the time, with a mean ranging from 5.39 days to 6.26 days [22,23,24]. Given that medication use was not addressed in our questionnaire, we are unable to say if patients were adhering to their medication schedules or not and whether depression was correlated with medication adherence.

With cautious interpretation, the exploratory multivariable analysis agrees with what has been reported, namely that receiving diabetes education has been found to lead to higher odds of mean days dedicated to self-care behavior. It has been suggested that the more knowledge and skills a patient has about the disease, the more confidence they will be able to muster in managing it [29]. Thus, it is important to include self-efficacy-improving strategies in healthcare providers’ diabetes interventions [13]. In addition to diabetes education, we found that a history of hospitalizations (OR: 1.81 [95% CI: 1.03 to 3.19, *p* = 0.039]) was positively associated with days spent on self-care activities. This might be partly explained by the fact that patients who experienced hospitalizations secondary to diabetes might become more conscious of the benefits of daily self-care activities to prevent further complications and hospitalizations. It is important to note, however, that other studies have found that hospitalizations are inversely correlated with self-care activities [30,31]. Furthermore, having asthma or other respiratory diseases was found to be associated with lower levels of self-care activities. However, it is worth noting that there is no reported literature on such an association, so we must be cautious about interpreting these results. In addition, the current study was not designed to assert the associations reported in Table 3 as this will require making assumptions about the data-generating process via DAGs and collecting more pertinent variables to test each variable of interest with the outcome of interest [16,32].

In our study, depressive symptoms were found to be prevalent as 54% of the patients had some level of depression and the median PHQ-9 score was five (IQR 2–8). Several studies of different ethnic groups have demonstrated a negative correlation between depression and self-care activities [8,12,25,26]. In a study by Gonzalez and colleagues, depression was associated with poorer adherence to diabetes self-care activities [12]. Also, studies in Turkey, Kuwait, and Saudi Arabia found a negative correlation between diabetes self-care activities and depression [26]. Al-Amer et al. found an indirect relationship between self-care activities and depression among Jordanians with diabetes [11]. Similarly, a Malaysian study did not find an association between self-care activities and depression [33]. Overall, such an association highlights the negative impact of different levels of depression on patients’ engagement in managing their health.

In addition to the association between the overall mean self-care activity score and depression, some components of self-care activities were negatively associated with depression when assessed individually. Patients who exercised were 14% less likely to have a lower PHQ-9 score. This finding further supports many studies that have found exercise to be an effective intervention for reducing depressive symptoms in individuals with type 2 diabetes, providing a potential pathway for improving mental well-being in this population [34]. Similarly, several interventional studies have demonstrated that adopting a healthy diet can improve the symptoms of depression in individuals with type 2 diabetes [35,36]. Our results seem to be consistent as those who followed a healthy diet were 11% less likely to have a lower PHQ-9 score. Therefore, the combination of a healthy diet and regular exercise may be beneficial in managing depression symptoms in individuals with type 2 diabetes.

The current study distinguishes itself from other studies by utilizing DAGs (directed acyclic diagrams) to illustrate the assumptions made about relationships. Previous studies performed simple correlation testing without adjustments for covariates [12,26]. Al-Amer (2016) utilized structural equation modeling (SEM) to investigate the interplay among several variables [11]. This approach provided a comprehensive assessment of how illness perception, depression, BMI, and glycosylated hemoglobin influence self-care activities by integrating multiple mediators. Notably, their model encompassed intriguing latent variables. They discerned an indirect relationship between depression and self-care activities, mediated by self-efficacy (β = −0.20, *p* = 0.003). In contrast, our study did not delve into such intricate interrelationships among variables, largely due to the constraints of our survey instruments. Our primary focus was on exploring the association between depression (treated as a dependent variable) and SDCA (as an independent variable). We operated under the premise that variations in SDCA could potentially influence depression levels in patients with diabetes. It is equally valid to question if the depression variable might impact SDCA. Given the study’s cross-sectional design, the temporal sequence of these variables was not established, hinting at the possibility of a bidirectional influence that was not examined in this study.

The use of the snowball sampling method via social media platforms was a limitation. While this nondiscriminatory, non-probabilistic approach facilitated access to individuals who are typically difficult to reach, it also potentially compromised the generalizability of our results. This is because the sampling technique can lead to an overrepresentation of certain demographic groups. In our study, there was an overrepresentation of the eastern region of Saudi Arabia (62.9% of the sample). With the cross-sectional survey design, there was also a chance for recall bias as the study was dependent on patients’ self-reporting. For instance, self-reporting might lead to an overestimation of adherence. That said, it is important to note that, in our study, the level of adherence reported was lower than in other studies. As mentioned earlier, our questionnaire did not include questions regarding adherence to medication schedules. For this reason, we did not establish any association between the PHQ-9 score and medication use. Moreover, we have not tested the possibility of latent variables that may mediate the effect between SDCA and depression. Furthermore, the multivariable analysis for the factors associated with diabetes self-care activities can be improved via collecting more unmeasured variables and drawing DAGs for each variable of interest. However, careful interpretation of the results in Table 3 is warranted as this may represent a classical Table 2 fallacy [32]. Although DAGs can help to identify the potential selection bias of covariates, they have their own limitations as well. First, they are limited to our previous knowledge of the subject matter. Therefore, several DAGs can be drawn if proper justification is provided. Secondly, DAGs do not provide insights into the strength or functional form of relationships (such as whether a relationship is additive or multiplicative). Thirdly, the representation of time ordering and feedback loops is challenging when using DAGs. Despite these limitations, DAGs are considered an efficient tool for communicating our underlying assumptions about the data generation process.

The current study identified valuable information regarding the level of depressive symptoms and the association between depression and daily self-care activities among the Saudi population. Increasing the number of daily self-care activities performed is associated with reduced depression levels, underscoring the need for targeted interventions. To advance research and healthcare practices, future studies should explore cultural influences and the efficacy of tailored interventions. Policy implications may include routine depression screening, integrated care models, public health campaigns, and professional training to enhance the overall well-being of individuals with type 2 diabetes in Saudi Arabia.

## 5. Conclusions

In conclusion, our study reveals a significant negative association between depression (PHQ-9 scores) and diabetes self-care activities (SDSCA). As daily self-care activities increase, depression levels decrease. The low adherence to self-care activities, particularly in the areas of exercise and foot care, emphasizes the need for tailored interventions. Our findings highlight the urgency of addressing depression, promoting self-care, and implementing targeted education strategies for individuals with type 2 diabetes in Saudi Arabia.

## Figures and Tables

**Table 1:** Participants’ demographic characteristics.

	Total (N = 252)	%
**Gender, n (%)**		
Female	114	45.2
**Age**		
18–34	39	15.5
35–54	104	31.3
55+	109	43.2
**Marital status, n (%)**		
Single	30	12.0
Married	204	81.3
Divorced	6	2.4
Widowed	11	4.4
**Place of residence, n (%)**		
Eastern province	158	62.9
Western region	60	23.9
Central region	18	7.2
Northern region	7	2.8
Southern region	8	3.2
**Education, n (%)**		
Illiterate	8	3.2
Elementary	4	1.6
Intermediate school	16	6.4
High school	38	15.1
Diploma	33	13.1
University	122	48.6
Postgraduate	30	12.0
**Employment status, n (%)**		
Student	15	6.0
Unemployed	45	17.9
Employed	91	36.3
Retired	100	39.8
**Health insurance, n (%)**		
Yes	130	52.0
No	120	48.0
**Smoker, n (%)**	191	76.0
**Duration of diabetes, n (%)**		
<5 years	85	32.5
5–10 years	60	23.8
>10 years	98	38.8
**Types of treatment, n (%)**		
No treatment	50	19.8
Insulin only	40	15.8
Injection (other than insulin)	5	1.9
Oral	104	41.2
Oral and insulin	49	19.4
**Comorbidities, n (%)**	239	94.5
**Diabetes complications, n (%)**	197	78.0
**Hospitalization, n (%)**	196	77.7
**Diabetes education, n (%)**	72	28.5

**Table 2:** Association of self-care activities and PHQ-9.

Self-Care Activities	Self-Care Activity Days	Association with PHQ-9 (OR with 95% CI, *p* Value)	Adjusted Association with PHQ-9 (OR with 95% CI, *p* Value)
Total score, median (IQR)	2.5 (1.4 to 3.5)	0.84 (0.74 to 0.97, *p* = 0.017 *)	0.83 (0.71 to 0.96, *p* = 0.013 *)
Diet, median days (IQR)	3.0 (0.5 to 5)	0.89 (0.82 to 0.98, *p* = 0.017 *)	0.94 (0.85 to 1.03, *p* = 0.045)
Blood glucose monitoring, median days (IQR)	2.5 (0 to 5.5)	0.98 (0.90 to 1.06, *p* = 0.574)	0.90 (0.82 to 0.99, *p* = 0.003 *)
Exercise, median days (IQR)	0.5 (0.0 to 3.0)	0.86 (0.77 to 0.95, *p* = 0.003 *)	0.88 (0.79 to 0.98, *p* = 0.002 *)
Foot care, median days (IQR)	0.5 (0.0 to 3.0)	0.97 (0.87 to 1.08, *p* = 0.600)	0.94 (0.84 to 1.01, *p* = 0.263)

An ordinal regression model was fitted to test the association between the self-care activity score and PHQ-9. In the adjusted estimates, we considered the total causal adjustments set to test the association. In this adjustment set, age, sex, social factors, insurance, diabetes duration, complications, smoking status, and comorbid conditions were included. PHQ-9: Patient Health Questionnaire. OR: Odds ratio. IQR: Interquartile range. * Statistically significant.

**Table 3:** Factors associated with diabetes self-care activities.

Predictor	Univariable Regression (OR with 95% CI, *p* Value)	Multivariable Regression (Adjusted OR with 95% CI, *p* Value)
Diabetes Onset	1.15 (0.89 to 1.48, *p* = 0.276)	1.09 (0.81 to 1.47, *p* = 0.538)
Diabetes Medications	1.08 (0.93 to 1.26, *p* = 0.285)	0.99 (0.83 to 1.19, *p* = 0.988)
Dyslipidemia	1.11 (0.70 to 1.76, *p* = 0.658)	1.05 (0.62 to 1.76, *p* = 0.847)
Hypertension	1.20 (0.76 to 1.89, *p* = 0.425)	1.33 (0.86 to 2.21, *p* = 0.262)
Other cardiovascular diseases	0.66 (0.27 to 1.59, *p* = 0.359)	0.81 (0.32 to 2.02, *p* = 0.659)
Asthma or other respiratory diseases	0.49 (0.21 to 1.14, *p* = 0.097)	0.40 (0.17 to 0.94, *p* = 0.035 *)
Chronic renal disease	2.06 (0.10 to 34.03, *p* = 0.630)	1.64 (0.06 to 34.21, *p* = 0.769)
Complications	0.79 (0.475 to 1.32, *p* = 0.377)	0.75 (0.42 to 1.34, *p* = 0.340)
Hospitalization	1.87 (1.12 to 3.16, *p* = 0.018 *)	1.81 (1.03 to 3.19, *p* = 0.039 *)
Diabetes education	2.00 (1.23 to 3.28, *p* = 0.006 *)	1.89 (1.09 to 3.29, *p* = 0.024 *)

* Statistically significant result.

## Data Availability

All the data of this research have been presented in this paper; however, the raw data are available upon request from the corresponding author (S.M.K.).
